# Cardiac resynchronization therapy through a subcutaneous tunnel assessed by phase analysis of gated myocardial perfusion SPECT imaging

**DOI:** 10.1007/s12350-022-02916-7

**Published:** 2022-03-08

**Authors:** Tadao Aikawa, Rui Kamada, Jiro Ogino, Toshinori Saitou, Naohiro Funayama, Daisuke Hotta

**Affiliations:** 1 Department of Cardiology, Hokkaido Cardiovascular Hospital, 1-30, Minami-27, Nishi-13, Chuo-ku, Sapporo, 064-8622 Japan; 2 Department of Radiology, Jichi Medical University Saitama Medical Center, 1-847 Amanuma-cho, Omiya-ku, Saitama, 330-8503 Japan; 3 Department of Cardiovascular Medicine, Faculty of Medicine and Graduate School of Medicine, Hokkaido University, Kita-15, Nishi-7, Kita-ku, Sapporo, 060-8638 Japan; 4 Department of Pathology, JR Sapporo Hospital, Kita-3, Higashi-1, Sapporo, 060-0033 Japan; 5 Department of Radiology, Hokkaido Cardiovascular Hospital, 1-30, Minami-27, Nishi-13, Chuo-ku, Sapporo, 064-8622 Japan

A 71-year-old woman was admitted to our hospital due to increasing dyspnea. She had hypothyroidism, bradycardia and atrial fibrillation, for which she received an MRI-incompatible pacemaker through the left subclavian vein at 54 years old. Echocardiography demonstrated a reduced left ventricular (LV) ejection fraction of 31%. Coronary angiography revealed no coronary abnormalities, whereas endomyocardial biopsy showed myocardial fibrosis (Figure 1A, B) with mucopolysaccharide accumulation detected by Alcian blue staining (Figure 1C), suggesting hypothyroidism induced cardiomyopathy.^1^ Despite the optimal medical therapy, her symptom remained New York Heart Association class III, and her LVEF was still reduced; therefore, she was considered for an upgrade to cardiac resynchronization therapy (CRT). Venography demonstrated total occlusion of the left brachiocephalic vein (Figure 2A). Given that intravascular lead extraction was considered high risk in this patient at the heart team meeting, we successfully implanted a quadripolar LV lead (Boston Scientific, Acuity^TM^ X4) through the right subclavian vein and tunneled it subcutaneously to the left pocket of the previous pacemaker under general anesthesia (Figure 2B, C, Video 1-2). Electrocardiography (ECG) after CRT implantation showed a shorter QRS duration than that before the procedure (Figure 3). Furthermore, impact of CRT on LV mechanical dyssynchrony was assessed by ECG-gated ^99m^Tc-sestamibi myocardial perfusion SPECT imaging with a dedicated phase analysis software (Heart Risk View-F; Nihon MediPhysics). Phase standard deviation and bandwidth histogram became narrow with an increase in LV ejection fraction and a decrease in LV volumes after CRT implantation (Figure 4, Video 3-4). She underwent ^18^F-fluorodeoxyglucose (^18^F-FDG) PET/CT after 20 hours fasting with low-carbohydrate diet preparation for screening of inflammatory heart disease, in which no ^18^F-FDG uptake was seen in the myocardium (Figure 2D).

**Figure 1:**
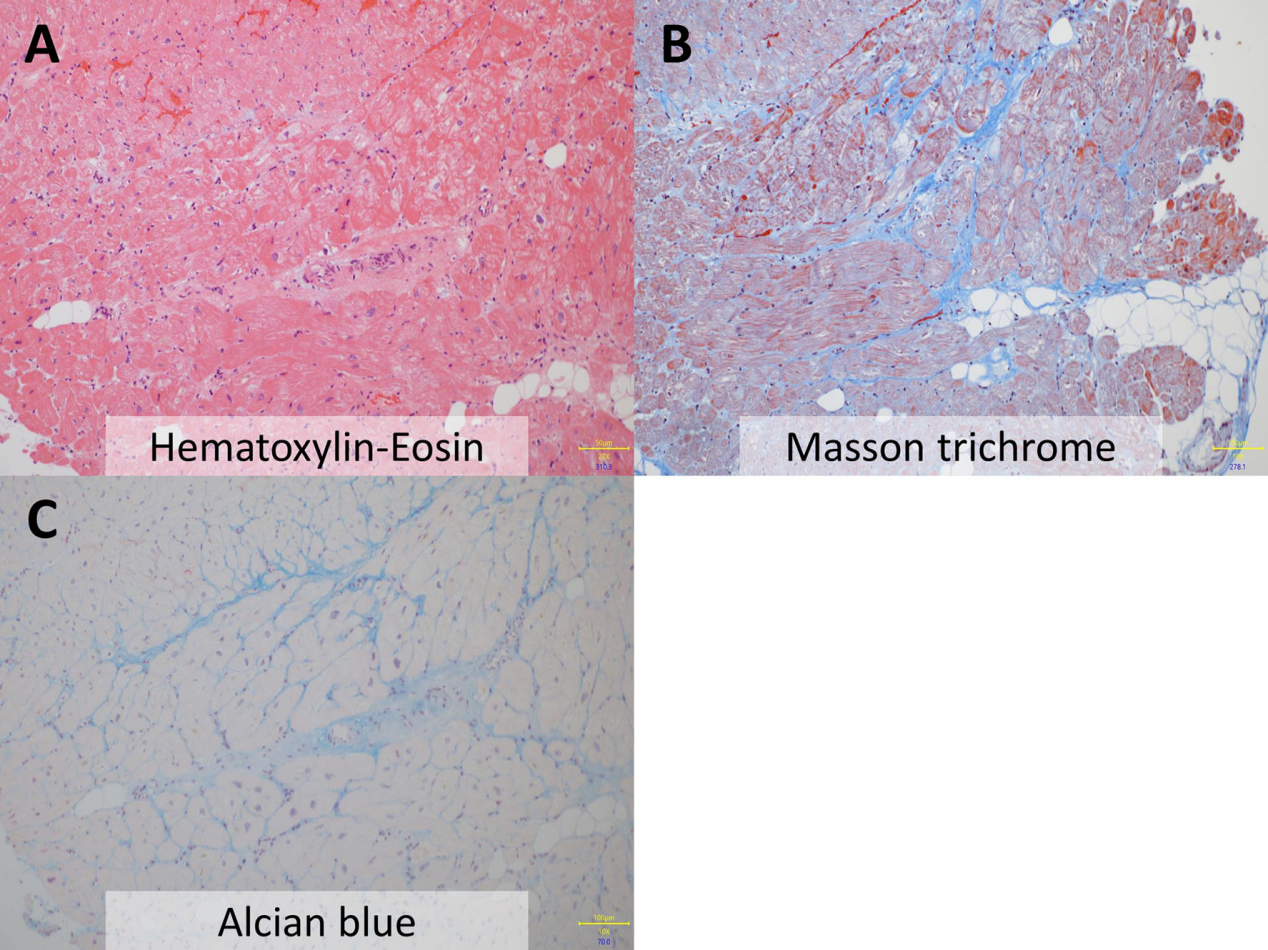
Histological sections obtained from right ventricle. Hematoxylin and eosin staining (**A**) and Masson trichrome staining (**B**) show mild myocyte hypertrophy and interstitial fibrosis with fatty infiltration. **C** Alcian blue staining shows the accumulation of mucopolysaccharides in the interstitium

**Figure 2:**
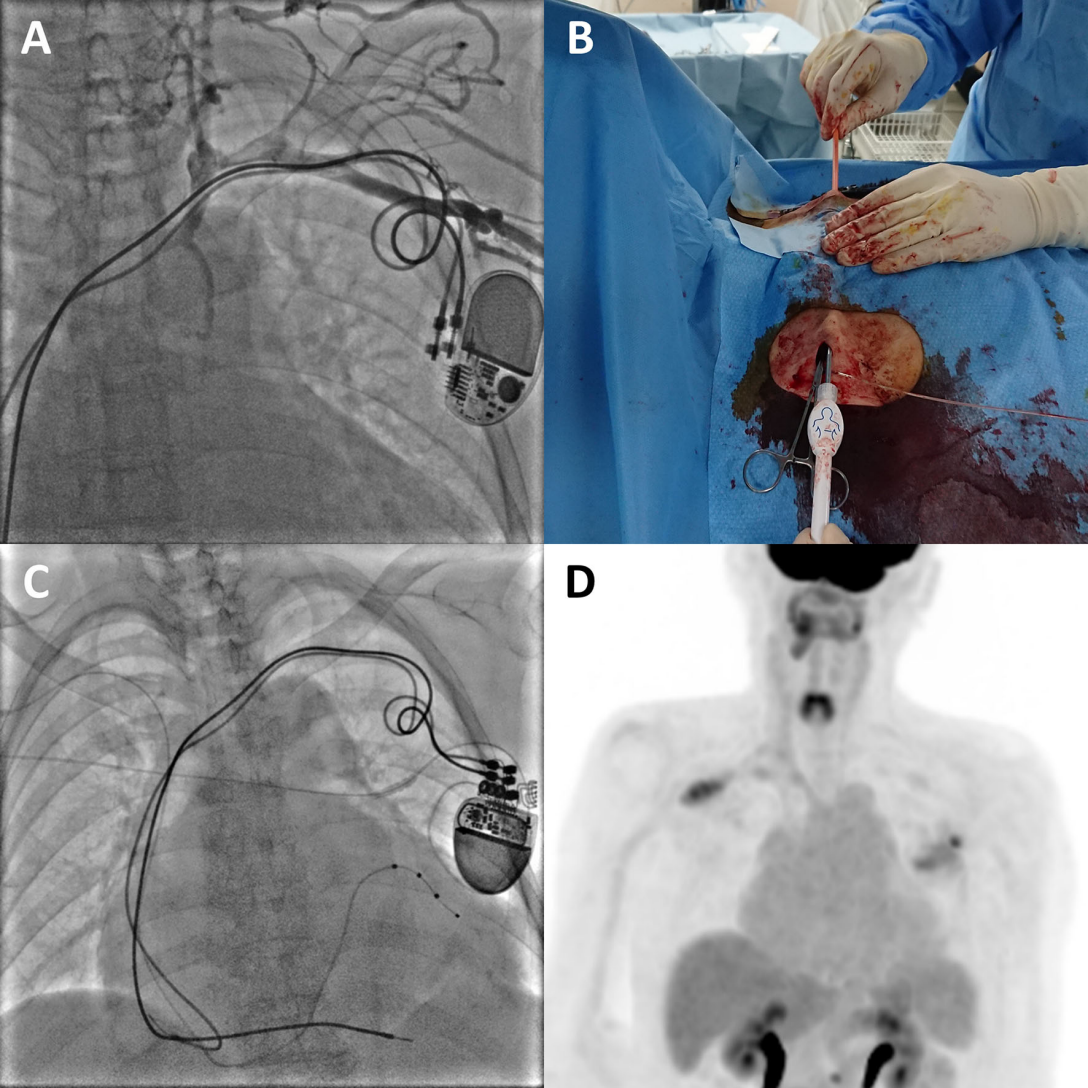
**A** Venography demonstrates total occlusion of the left brachiocephalic vein with collateral flow drained into the azygos system. **B** Subcutaneous tunneling of the left ventricular lead from the right to the left pocket of the previous pacemaker. **C** Chest X-ray after cardiac resynchronization therapy. **D** ^18^F-fluorodeoxyglucose (^18^F-FDG) positron emission tomography/computed tomography after 20 hours fasting with low-carbohydrate diet preparation shows no ^18^F-FDG uptake in the myocardium. Focal ^18^F-FDG uptake is seen in both subclavian wounds after cardiac resynchronization therapy

**Figure 3:**
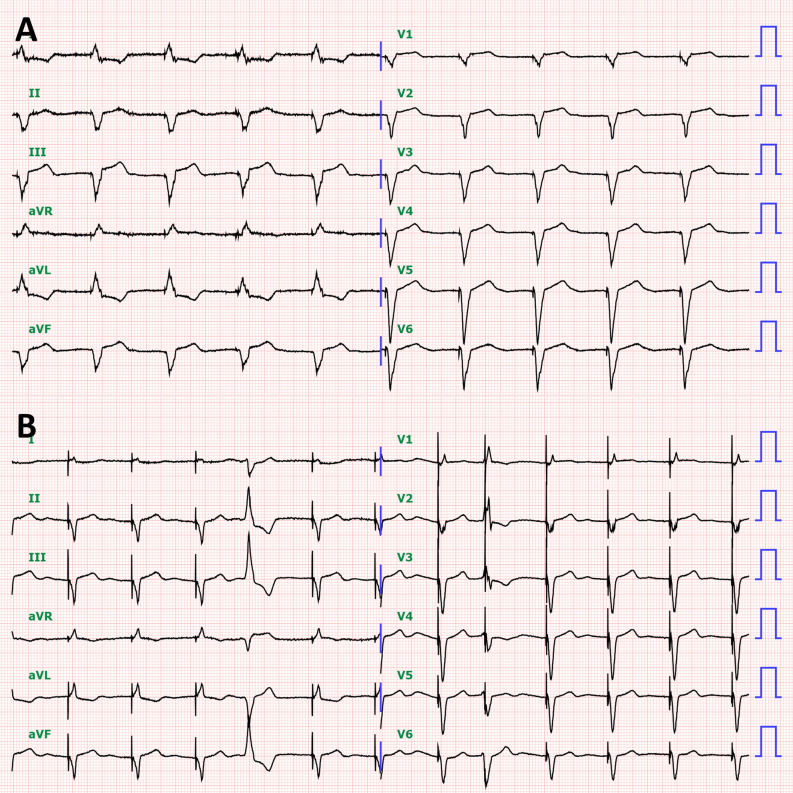
Electrocardiography (ECG) before (**A**) and after cardiac resynchronization therapy (**B**). ECG after cardiac resynchronization therapy shows a shorter QRS duration than that before the procedure

**Figure 4:**
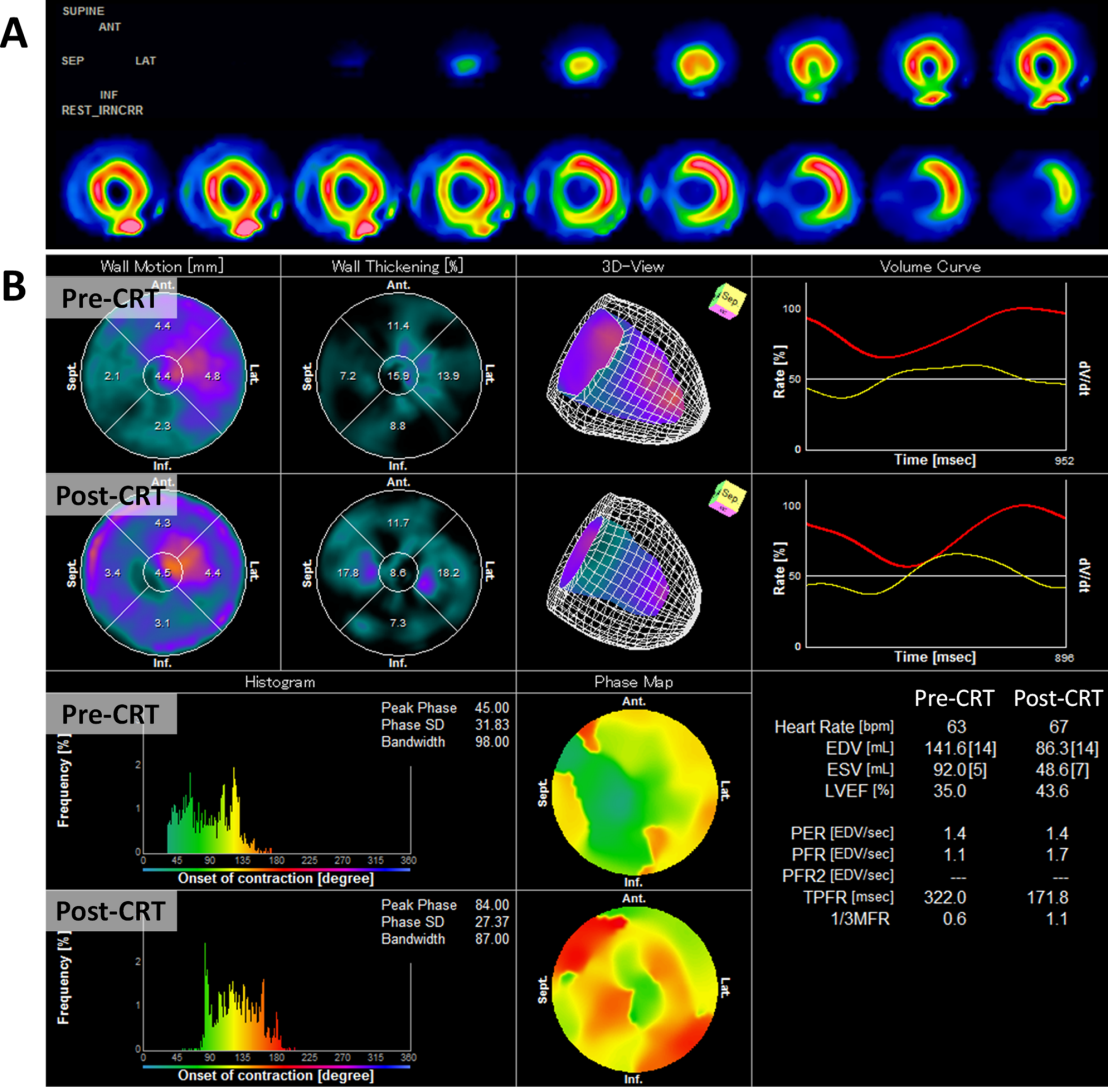
**A** ^99m^Tc-sestamibi myocardial perfusion SPECT imaging before cardiac resynchronization therapy (CRT). **B** Phase analysis on ECG-gated SPECT imaging before and after CRT. Phase standard deviation (SD) and bandwidth histogram become narrow with an increase in LV ejection fraction (from 35.0% to 43.6%) and a decrease in LV volumes after CRT implantation

Although LV dyssynchrony can be analyzed using different imaging modalities,^2^ nuclear imaging is feasible even in patients with MRI-incompatible devices and a reasonable approach to evaluating myocardial scar burden and changes in LV volumes and dyssynchrony after CRT in these patients.

## Supplementary Information

Below is the link to the electronic supplementary material.

Supplementary file1 **Video 1.** Making a subcutaneous tunnel from the left pocket of the previous pacemaker to the right subclavian one. (MP4 6842 kb)

Supplementary file2 **Video 2.** Placing the left ventricular lead through the subcutaneous tunnel. (MP4 867 kb)

Supplementary file3 **Video 3.** Gated SPECT before cardiac resynchronization therapy. (MP4 715 kb)

Supplementary file4 **Video 4.** Gated SPECT after cardiac resynchronization therapy. (MP4 682 kb)
